# Genome-Wide Identification of *Serine carboxypeptidase-like* (*SCPL*) Genes in Rice and Their Expression in Response to *Rhizoctonia solani*

**DOI:** 10.3390/ijms27219850

**Published:** 2026-11-03

**Authors:** Yu Wang, Shiji Feng, Chuan Chen, Jinlong Xue, Shuwei Zhang, Ting He, Guimei Guo, Longhua Zhou, Chenghong Liu, Zhiwei Chen

**Affiliations:** 1 Shanghai Key Laboratory of Agricultural Genetics and Breeding (21DZ2271900), Biotechnology Research Institute of Shanghai Academy of Agricultural Sciences, Shanghai 201106, China; yuwang@saas.sh.cn (Y.W.);; 2 Shanghai Pudong New Area Agricultural Technology Extension Center, Shanghai 201201, China

**Keywords:** genome-wide identification, gene family, rice, serine carboxypeptidase-like protein, sheath blight

## Abstract

Serine carboxypeptidase-like (SCPL) proteins play important roles in plant secondary metabolism, growth, and stress responses. Several SCPL members have been reported to participate in the biosynthesis of antimicrobial compounds associated with plant immunity. However, systematic characterization of the *SCPL* gene family in rice and its potential involvement in sheath blight (ShB) responses remains largely unclear. In this study, 45 *SCPL* genes were identified using the IRGSP-1.0 reference assembly and Ensembl Plants release 54 annotation and designated as *OsSCPL1*–*OsSCPL45*. Phylogenetic analysis of SCPL proteins from rice, Arabidopsis, soybean, and maize classified these proteins into three groups. Gene structures, conserved motifs, chromosomal distribution, and promoter *cis*-elements were subsequently analyzed. Chromosomal mapping and collinearity analysis revealed that both tandem and segmental duplications contributed to the expansion of the *OsSCPL* gene family. Promoter analysis identified numerous *cis*-elements associated with stress and hormone signaling. Mock-controlled RT-qPCR results of *OsSCPL* genes in resistant rice genotype YSBR1 and susceptible rice genotype Lemont (LE) showed that 14 *OsSCPL* genes were significantly up- or downregulated following *Rhizoctonia solani* inoculation in at least one genotype. The segmentally duplicated gene pair *OsSCPL4* was significantly upregulated in both genotypes, whereas *OsSCPL25* was significantly upregulated in YSBR1 but did not reach significance in LE. This study provides an updated genomic framework for the *SCPL* gene family in rice and suggests potential roles for *OsSCPL4* and *OsSCPL25* in rice responses to *R. solani*, laying a foundation for further functional studies.

## 1. Introduction

Serine carboxypeptidase-like (SCPL) proteins, belonging to the S10 subgroup of the α/β hydrolase superfamily, are widely implicated in protein turnover and C-terminal processing, thereby playing essential roles in plant growth, development, and stress responses [1,2,3]. Notably, a specialized lineage within this family has undergone functional divergence, evolving catalytic activity as acyltransferases rather than classical proteases [4,5]. This SCPL-mediated acylation significantly enhanced the structural diversity and biological activity of plant secondary metabolites. Based on these functional and phylogenetic relationships, SCPL proteins in higher plants are typically classified into three subgroups (I, II, and III).

Accumulating evidence shows that *SCPL* genes are involved in plant responses to environmental stresses. In oat, *Sad7*/*SCPL1* encodes an acyltransferase required for avenacin acylation, and its loss increases susceptibility to fungal pathogens [6]. In cotton, silencing *Gh_SCPL42* increases susceptibility to Verticillium wilt and reduces the expression of several salicylic acid (SA)- and jasmonic acid (JA)-related genes [7]. *SCPL* genes have also been associated with abiotic stress responses. Multiple *SCPL* genes in soybean were responsive to diverse abiotic stimuli [8], while wheat *TaSCPL184-6D* was found to improve drought and salt tolerance when heterologously expressed in *Arabidopsis* [9]. A tomato *SCP* gene was identified as a member of the late wound-inducible genes and was induced by wounding, systemin and methyl jasmonate [10]. These studies indicate that SCPL family members play roles in plant defense and responses to biotic and abiotic stresses [4,5,6,7,8,9,10,11].

As one of the most important staple crops worldwide, rice is severely threatened by sheath blight (ShB), a devastating disease caused by the fungus *Rhizoctonia solani* that can result in substantial yield losses, particularly under high-input production systems [12]. In rice, the *SCPL* gene *OsBISCPL1* was induced by blast infection and by defense-related signals, including salicylic acid, jasmonic acid, and the ethylene precursor 1-aminocyclopropane-1-carboxylic acid (ACC) [13]. These signals are also relevant to sheath blight defense; for example, salicylic acid-dependent immunity contributed to rice resistance against *R. solani* [14]. The responsiveness of *OsBISCPL1* to pathogen infection and defense-related signals therefore provides a basis for investigating whether other rice *SCPL* genes respond to *R. solani*. However, their expression responses to this pathogen and their possible contributions to ShB resistance remain insufficiently characterized.

Although the rice *SCPL* gene family has previously been identified [15,16], reassessment using a clearly specified genome assembly and annotation release was needed to reassess the family membership. In this study, the rice *SCPL* gene family was re-identified using the IRGSP-1.0 genome assembly and Ensembl Plants release 54 annotation. The expression patterns of *OsSCPL* genes in response to *R. solani* infection were further investigated in resistant rice genotype YSBR1 and susceptible rice genotype Lemont (LE). This study provides a genomic framework and expression-based evidence to guide subsequent functional investigations.

## 2. Results

### 2.1. Identification and Physicochemical Analysis of OsSCPL Gene Family Members in Rice

Previous studies reported 71 putative *SCPL* genes in rice [15,16]. To update the identification of this gene family, we conducted a new genome-wide search using the *Oryza sativa* ssp. *japonica* IRGSP-1.0 reference assembly, Ensembl Plants release 54 annotation, and a two-step strategy combining BLASTP similarity searches with HMMER screening against the PF00450 profile. A total of 45 proteins satisfied both identification criteria and were retained, all of which showed significant PF00450 (peptidase S10) matches (Supplementary Table S2). Based on these identification and domain-validation results, 45 *OsSCPL* genes were retained and designated *OsSCPL1–OsSCPL45* according to their chromosomal positions. Their current stable locus identifiers and correspondence with previously reported candidates are provided in Supplementary Table S3. For subsequent phylogenetic and comparative analyses, the dataset also included 49 *SCPL* genes from *Zea mays*, 68 from *Glycine max*, and 51 from *Arabidopsis thaliana*, resulting in a total of 213 *SCPL* genes across the four species. Their genomic and predicted protein characteristics are summarized in Supplementary Table S4. Comparison with the 71 previously reported rice *SCPL* candidates showed that 49 candidates corresponded to 39 current stable loci retained in the present gene set, including cases in which multiple previous candidates mapped to the same locus. The remaining 22 candidates had no retained counterpart under the identification criteria used in this study (Supplementary Table S5).

The 45 OsSCPL proteins ranged from 307 to 524 amino acids in length, with predicted molecular weights (MolWt) of 34.19–57.98 kDa and theoretical isoelectric points (pI) ranging from 4.52 to 9.36, with 32 members classified as acidic (pI < 7) and 13 as basic (pI > 7). The complete predicted physicochemical properties and predicted subcellular localization results of individual OsSCPL proteins are provided in Supplementary Table S4.

### 2.2. Analysis of Domain Composition and Conserved Motifs of OsSCPL Family Proteins

**Figure 1:**
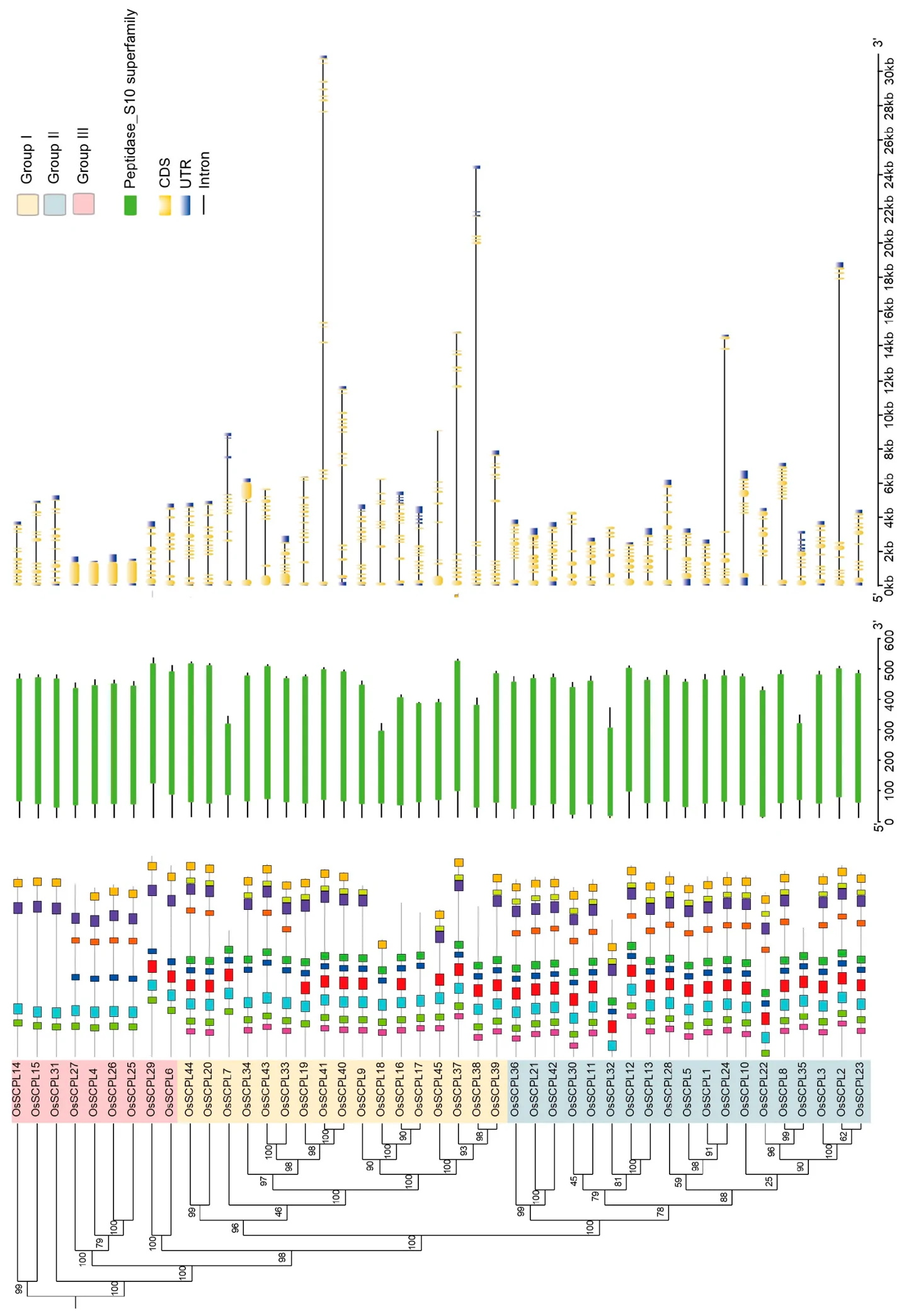
Phylogenetic tree, gene structure and conserved motif analysis of the *OsSCPL* family. From left to right, the panels show the phylogenetic tree, conserved motif distribution, protein domain organization, and exon–intron structures. Ten putative motifs are indicated as colored boxes. Introns and exons are indicated by black lines and orange boxes, respectively. The lengths of introns, exons and proteins of each gene can be inferred using the scale displayed at the bottom.

The gene structures of *OsSCPL* members were analyzed, including exons, introns, and annotated 5′ and 3′ untranslated regions (UTR) (Figure 1). Most *OsSCPL* genes contained 9 to 13 exons. In contrast, *OsSCPL4*, *OsSCPL25*, *OsSCPL26*, and *OsSCPL27* each had only a single exon, while *OsSCPL34* contained three exons. Based on the representative transcript annotations, no 5′ or 3′ UTR was annotated for *OsSCPL32*, *OsSCPL43*, or *OsSCPL45*. Only a 3′ UTR was annotated for *OsSCPL12*, *OsSCPL35*, and *OsSCPL37*, whereas only a 5′ UTR was annotated for *OsSCPL30*. Both 5′ and 3′ UTRs were annotated for the remaining genes.

Conserved motif analysis using MEME showed that motif composition and arrangement were generally conserved across the family, although partial motif loss was observed in specific members. All 45 members contained the conserved PF00450 (peptidase S10) domain.

### 2.3. Phylogenetic Tree and Chromosome Mapping of OsSCPL Gene Family

To investigate evolutionary relationships of SCPL proteins, a phylogenetic tree was constructed using full-length SCPL protein sequences from *Oryza sativa* (rice), *Arabidopsis thaliana*, *Glycine max* (soybean), and *Zea mays* (maize).

**Figure 2:**
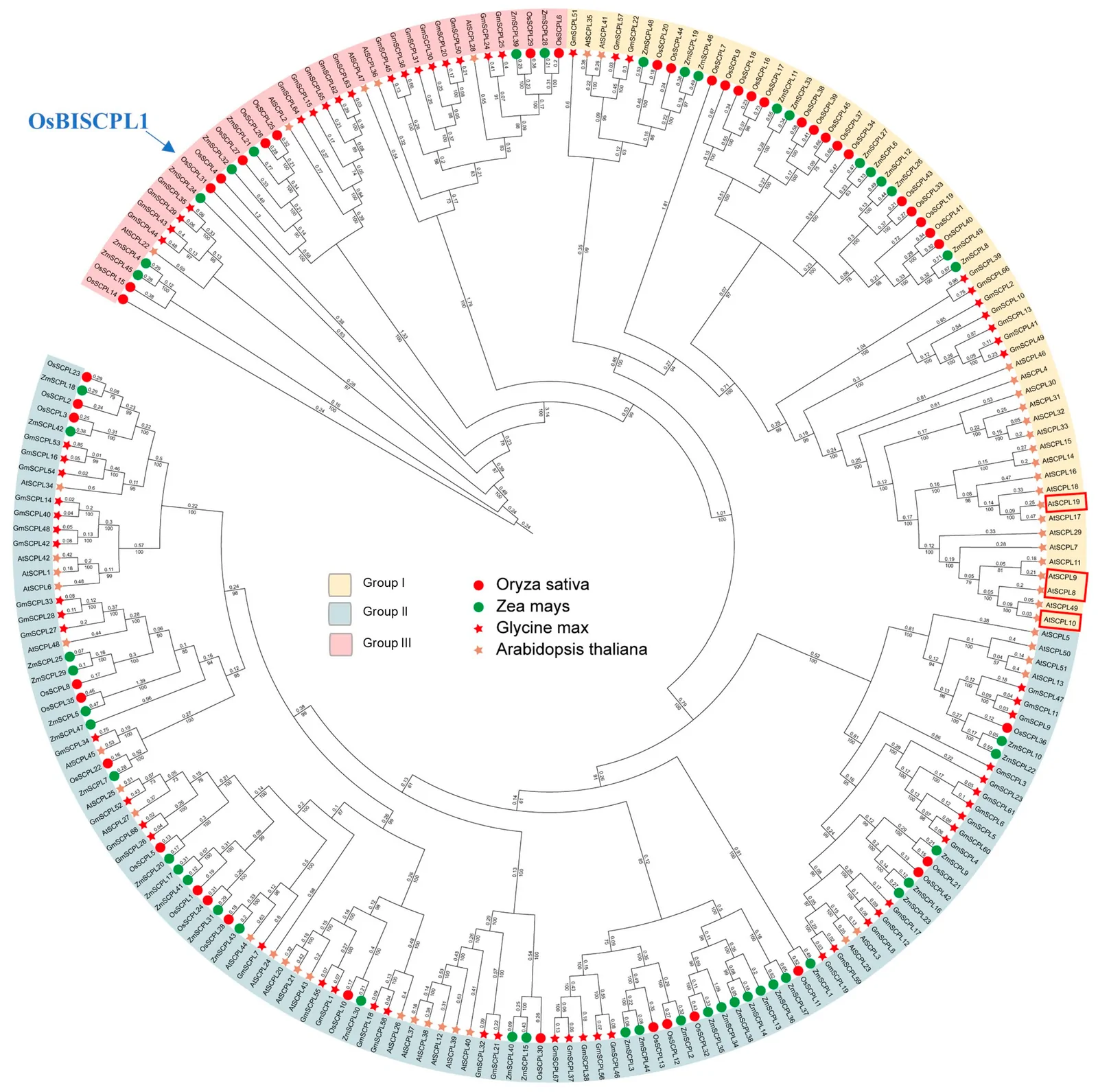
Phylogenetic tree of the *SCPL* gene family in *Oryza sativa* (rice), *Zea mays* (maize), *Glycine max* (soybean), and *Arabidopsis thaliana*. The phylogenetic tree was divided into three groups: Group I (yellow), Group II (blue), and Group III (pink). Different symbols represent SCPL proteins from *Oryza sativa* (red circles), *Zea mays* (green circles), *Glycine max* (red stars), and *Arabidopsis thaliana* (orange stars). Four experimentally characterized Arabidopsis SCPL acyltransferases, SNG1/SMT (AtSCPL8), SST (AtSCPL9), SAT (AtSCPL10), and SCT (AtSCPL19), were highlighted with red boxes.

**Figure 3:**
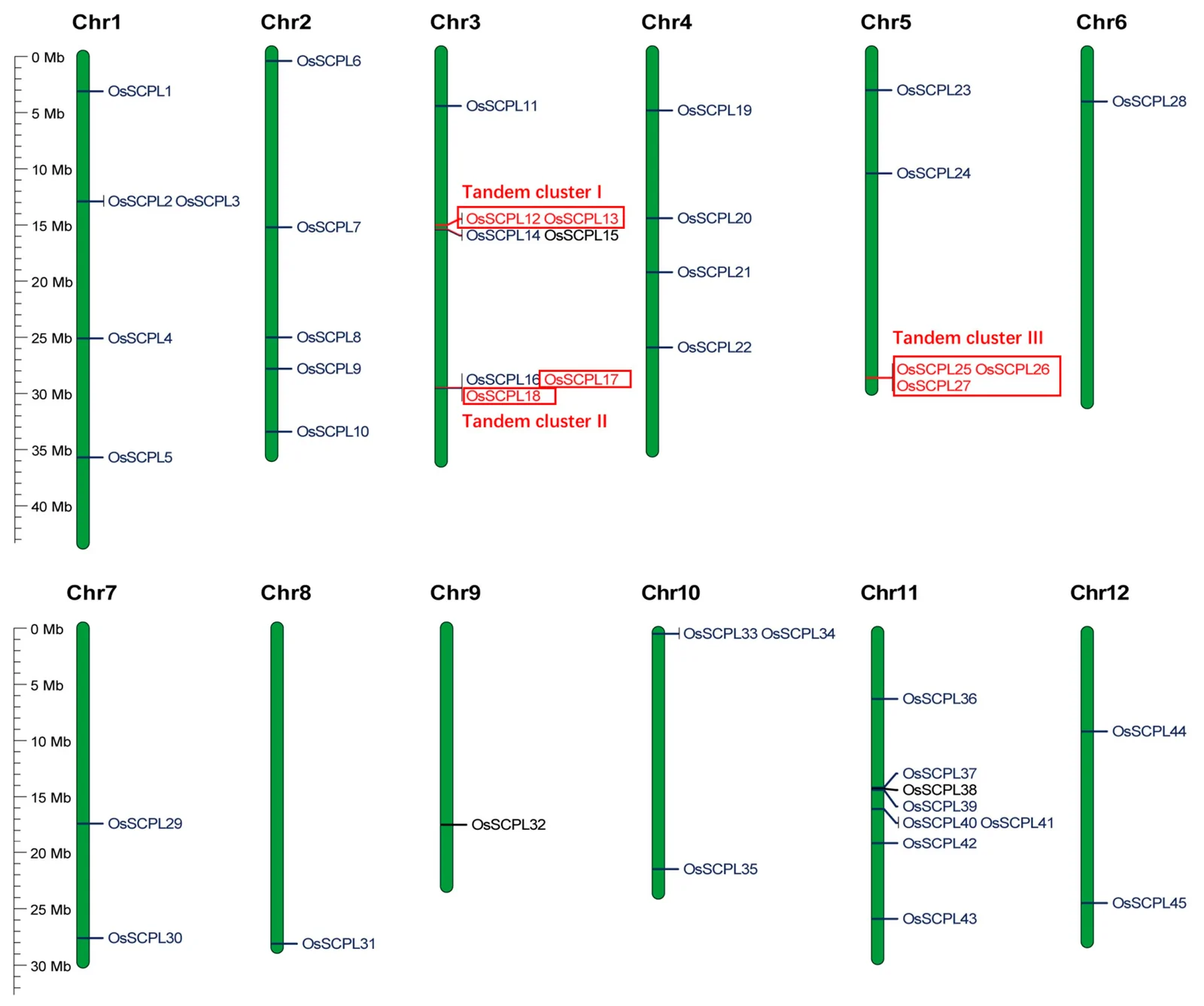
Chromosomal distribution of the *SCPL* gene family in rice. Red boxes indicate the three tandem duplication clusters, defined as homologous genes directly adjacent on the same chromosome with no intervening protein-coding genes.

As shown in Figure 2, a total of 213 SCPLs from the four species were classified into three distinct groups: Group I, Group II, and Group III. Notably, the four functionally characterized Arabidopsis SCPL acyltransferases, SNG1/SMT (AtSCPL8) [4], SST (AtSCPL9) [2], SAT (AtSCPL10) [2], and SCT (AtSCPL19) [3], all clustered within Group I and were highlighted with red boxes. Chromosomal mapping showed that the 45 *OsSCPL* genes were unevenly distributed across the 12 rice chromosomes, with the highest density observed on Chr3 and Chr11 (Figure 3).

### 2.4. Collinearity Analysis of the SCPL Gene Family

Gene duplication events are primary drivers of gene family expansion and functional evolution. We investigated the expansion mechanisms of the *OsSCPL* family based on chromosomal location and sequence homology. Internally, two distinct types of duplication were identified.

Tandem duplication was defined as homologous genes immediately adjacent on the same chromosome, with no intervening genes in the analyzed protein-coding gene set. In total, three tandem duplication clusters were identified including *OsSCPL12*–*OsSCPL13* and *OsSCPL17*–*OsSCPL18* on Chr3, and *OsSCPL25*–*OsSCPL26*–*OsSCPL27* on Chr5 (Figure 3).

**Figure 4:**
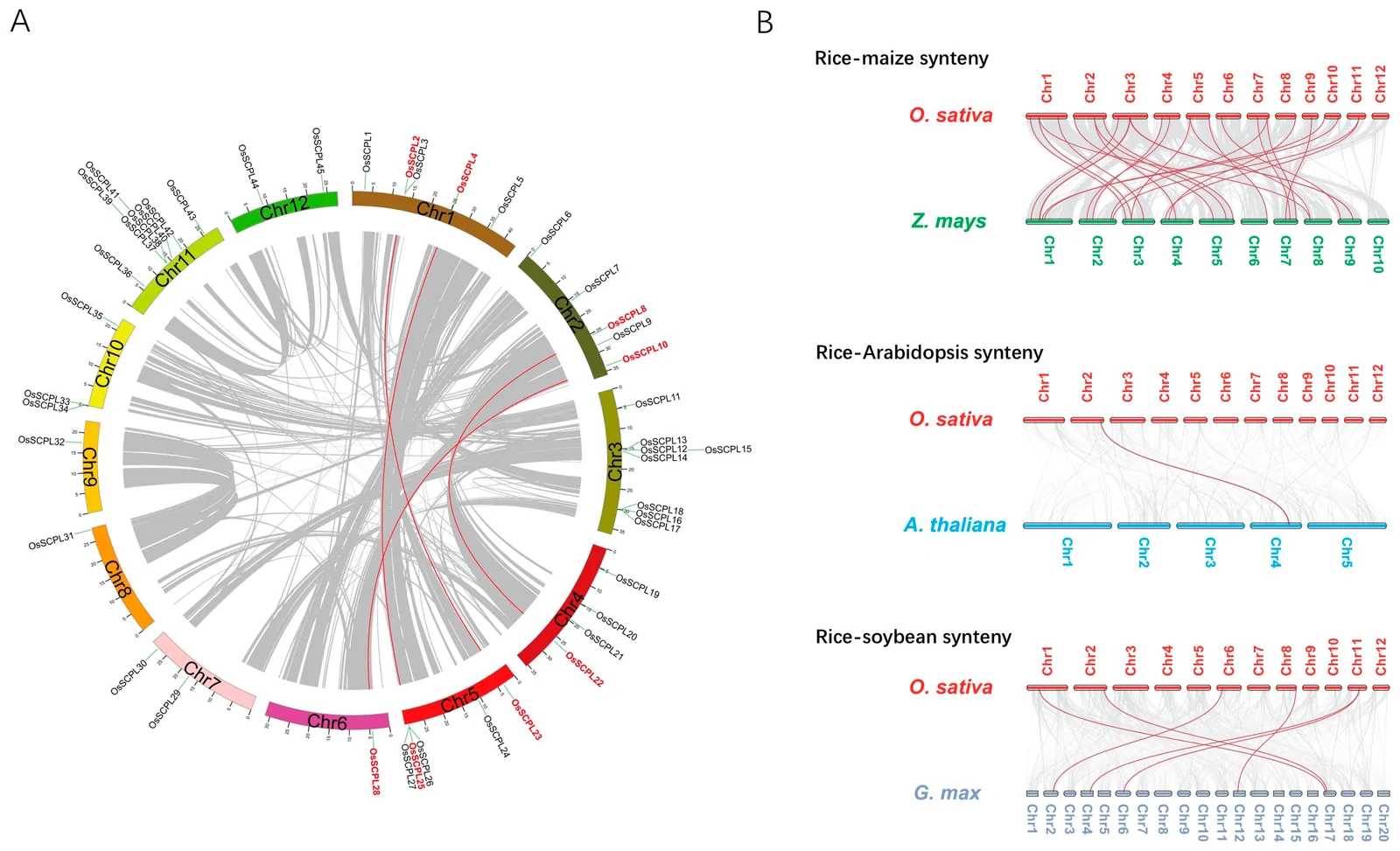
Collinearity and synteny analysis of the *OsSCPL* gene family. (**A**) Collinearity relationship of *SCPL* gene family members in rice. Red lines indicate collinear blocks of *SCPL* genes. (**B**) Syntenic analysis of *SCPL* genes in *Oryza sativa*, *Arabidopsis thaliana*, *Glycine max*, and *Zea mays*. Chromosomes are labeled as Chr1, Chr2, etc. Gray lines in the background indicate the collinear blocks within rice and other plant genomes, whereas red lines highlight syntenic *SCPL* gene pairs.

The second type is segmental duplication, which comprises four specific gene pairs located on different chromosomes, namely *OsSCPL2*–*OsSCPL23*, *OsSCPL4*–*OsSCPL25*, *OsSCPL8*–*OsSCPL22*, and *OsSCPL10*–*OsSCPL28* (Figure 4A). These findings suggest that both tandem and segmental duplications have contributed to the *OsSCPL* family expansion.

The Ka, Ks, and Ka/Ks values for the analyzed *OsSCPL* gene pairs are provided in Supplementary Table S6. All calculated Ka/Ks ratios were below 1, ranging from 0.0029 to 0.3955, a pattern consistent with purifying selection acting on the coding sequences of these gene pairs. The *OsSCPL4*/*OsSCPL25* pair had Ka and Ks values of 0.3071 and 2.8515, respectively, yielding a Ka/Ks ratio of 0.1077.

To further investigate the evolutionary history of *SCPL* genes in rice, we conducted a comparative synteny analysis between rice and three other plant species: *Arabidopsis thaliana*, *Glycine max* (soybean), and *Zea mays* (maize) (Figure 4B). We identified 28 syntenic gene pairs between rice and maize, whereas only 6 and 2 pairs were found in soybean and *Arabidopsis*, respectively. The greater number of syntenic gene pairs between rice and maize is consistent with their closer evolutionary relationship as monocot species [17].

### 2.5. Analysis of Cis-Acting Elements in the Promoter of OsSCPL Gene Family

**Figure 5:**
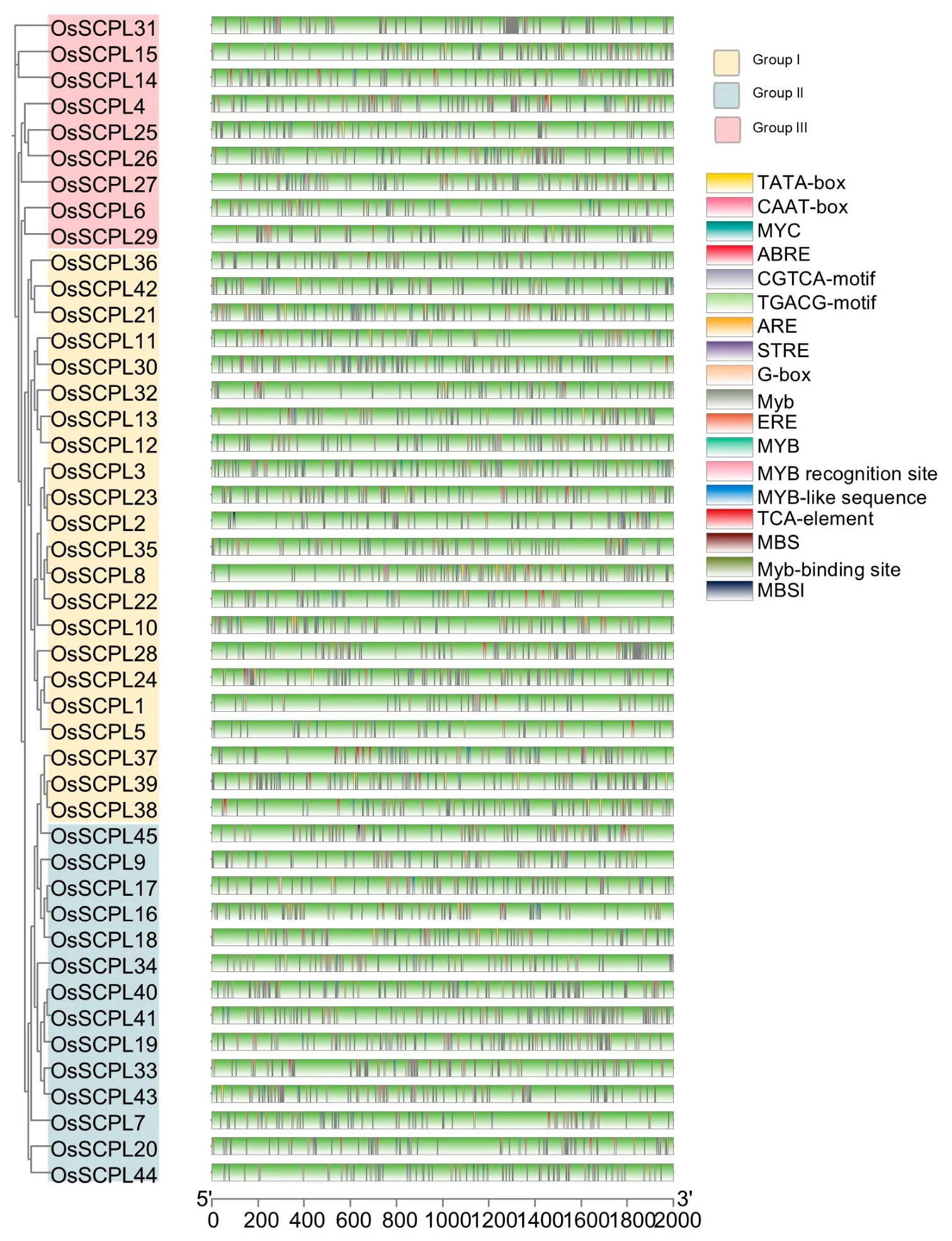
*Cis*-acting element analysis of the *SCPL* gene family.

PlantCARE analysis of the 2000-bp upstream regions predicted that *OsSCPL* promoters contain putative hormone- and stress-responsive *cis*-regulatory motifs (Figure 5, Supplementary Table S7).

MYC and abscisic acid-responsive element (ABRE) motifs were predicted in 42 and 41 promoters, respectively. The methyl jasmonate-responsive CGTCA-motif and TGACG-motif were each detected in 41 promoters. Putative stress-responsive motifs, including STRE and ARE, were detected in 38 and 35 promoters, respectively. MYB- and MYB-binding site (MBS)-related motifs were also widely distributed among the *OsSCPL* promoters.

The predicted motif composition varied among *OsSCPL* promoters. Ethylene-responsive element (ERE), TCA-element, and TGA-element were detected in 23, 18, and 19 promoters, respectively. In addition, dehydration-responsive element (DRE) elements related to drought and salt responsiveness were detected in 14 genes, and gibberellin-responsive elements such as the P-box and gibberellin-responsive element motif (GARE-motif) were identified in 11–13 genes. These results indicate that different *OsSCPL* genes contain distinct combinations of regulatory elements in their promoter regions.

### 2.6. Differential Expression of OsSCPL Gene Family Members Under R. solani Inoculation

**Figure 6:**
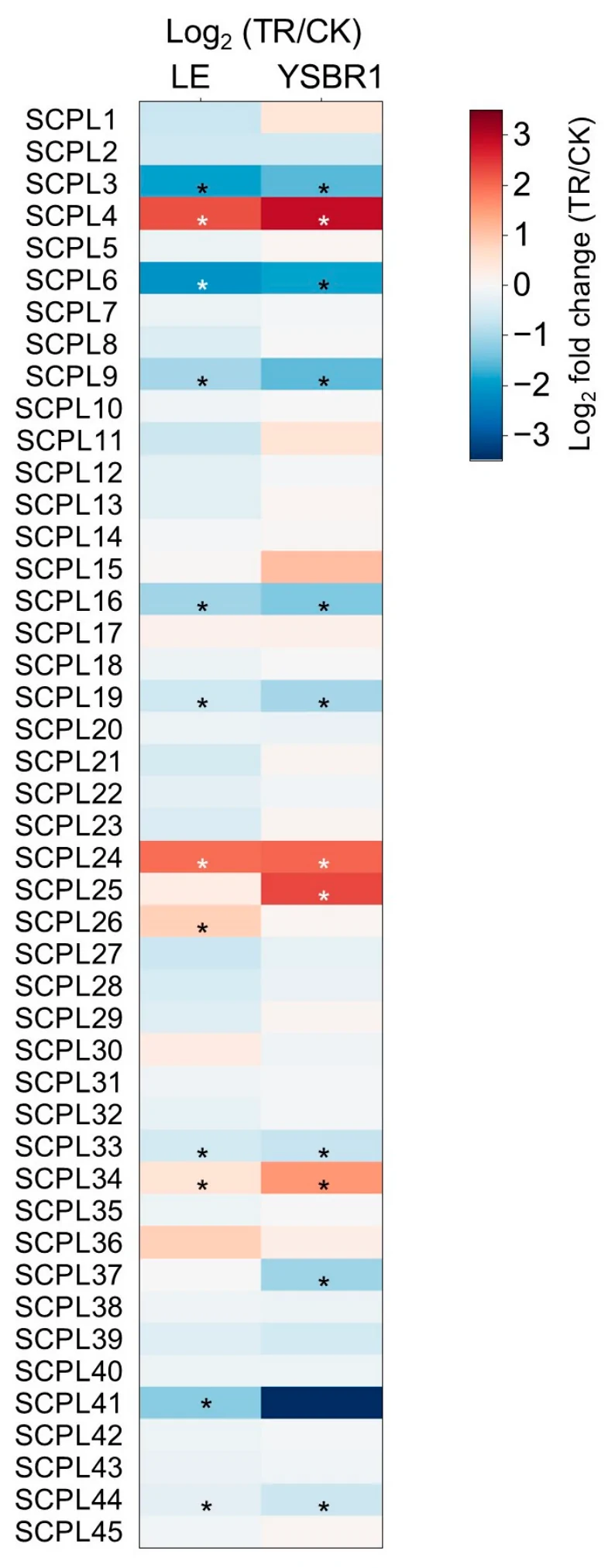
Expression profiles of 45 *OsSCPL* genes in LE and YSBR1 at 24 h after *Rhizoctonia solani* inoculation. Heatmap values represent log_2_(TR/CK), calculated from RT-qPCR data using the 2^−^ΔΔCt method with *ACTIN* as the reference gene. TR represents *R. solani*-inoculated samples, and CK represents time-matched mock-treated samples. Three independent biological replicates were analyzed per group. Asterisks within the heatmap cells indicate nominal *p* < 0.05 for comparisons between TR and time-matched CK within each genotype, determined using two-sided Welch’s *t*-tests on ΔCt values. These within-genotype comparisons were not adjusted for multiple testing.

To characterize the expression responses of *OsSCPL* genes to *R. solani* inoculation, all 45 genes were examined by RT-qPCR in the resistant rice genotype YSBR1 and the susceptible genotype LE at 24 h after inoculation. Time-matched mock-inoculated plants were used as controls (Figure 6, Supplementary Table S8). Fourteen genes showed significant expression differences between inoculated and mock-treated samples in at least one genotype based on unadjusted *p* < 0.05.

*OsSCPL4*, *OsSCPL24*, and *OsSCPL34* were significantly upregulated in both genotypes, whereas *OsSCPL3*, *OsSCPL6*, *OsSCPL9*, *OsSCPL16*, *OsSCPL19*, *OsSCPL33* and *OsSCPL44* were significantly downregulated in both genotypes. *OsSCPL26* was significantly upregulated in LE but not in YSBR1, whereas *OsSCPL41* was significantly downregulated in LE but not in YSBR1. In contrast, *OsSCPL25* was significantly upregulated and *OsSCPL37* was significantly downregulated in YSBR1, while neither gene showed a significant expression change in LE. These findings identify candidate *OsSCPL* genes responsive to *R. solani* inoculation for further functional investigation.

## 3. Discussion

In this study, 45 *OsSCPL* genes were identified using the *Oryza sativa* ssp. *japonica* IRGSP-1.0 reference assembly and Ensembl Plants release 54 annotation, fewer than the 71 candidates reported previously [15,16]. Comparison with the previous dataset showed that 49 of the 71 candidates corresponded to 39 current stable loci retained in the present gene set, including cases in which multiple previous candidates mapped to the same locus. The remaining 22 candidates had no retained counterpart under the identification criteria used in this study. Conversely, six loci retained in the current gene set were not represented in the previous dataset. These differences likely reflect updates to rice gene models and locus identifiers, together with the use of a uniform identification procedure. All 45 retained proteins showed significant PF00450 matches in the standardized HMMER re-evaluation. The correspondence between the previous candidates and the current gene set is provided in Supplementary Tables S3 and S5.

Gene duplication is an important force in the expansion and functional diversification of plant gene families. Our results showed that both tandem and segmental duplication events contributed to the expansion of the *OsSCPL* family. Tandemly duplicated genes were mainly organized in clusters on specific chromosomes, whereas segmentally duplicated gene pairs were distributed across different genomic regions (Figure 3 and Figure 4). Such duplication events can provide genetic material for subsequent functional divergence and adaptation to environmental changes [18,19]. All analyzed duplicate pairs had Ka/Ks ratios below 1, consistent with purifying selection. This suggests that mutations that could impair protein function were less likely to be retained during evolution, helping preserve important features of OsSCPL proteins.

The family also showed variation in gene structure and predicted promoter composition. Most members contained multiple exons, whereas *OsSCPL4*, *OsSCPL25*, *OsSCPL26*, and *OsSCPL27* each contained a single exon. Despite their shared exon structure, these four genes showed different combinations of predicted promoter elements, identifying promoter regulation as a direction for investigating their expression patterns. Across the family, TGA-element, GARE-motif, and Myb-binding site occurred in higher proportions of inoculation-responsive gene promoters than in promoters of genes without significant expression changes (57.1% versus 35.5%, 42.9% versus 22.6%, and 71.4% versus 41.9%, respectively; Supplementary Tables S7 and S8). This pattern raises the possibility that growth-related hormone signals and MYB-mediated regulation contribute to the transcriptional responses of some *OsSCPL* members during infection.

A notable example is the duplicated gene pair *OsSCPL4*/*OsSCPL25*. At 24 h after inoculation, *OsSCPL4* was significantly induced in both LE and YSBR1, whereas *OsSCPL25* was significantly induced in YSBR1 but not in LE. These results show that the two duplicated genes had different expression responses under the conditions examined. Further evidence is required to determine whether they have undergone regulatory or functional divergence.

Other *OsSCPL* members also showed pathogen-dependent responses. For example, overexpression of *OsSCPL31*, previously reported as *OsBISCPL1* [13], enhanced resistance to *Pseudomonas syringae* and *Alternaria brassicicola* in *Arabidopsis*, whereas *OsSCPL31* was not significantly induced by *R. solani* in the present study. This difference may reflect variation in pathogen type, host background, tissue, or sampling time. Previous studies have shown that JA and SA signaling, as well as secondary metabolites including flavonoids and diterpenoid phytoalexins, are involved in rice responses to *R. solani* [14]. The phylogenetic relationship of *OsSCPL4* and *OsSCPL25* with *OsBISCPL1*, together with their expression responses after inoculation, supports further investigation of their possible roles in defense-related signaling and secondary metabolism.

## 4. Materials and Methods

### 4.1. Plant Material

YSBR1, a *japonica*/*indica* hybrid developed through pedigree breeding, demonstrates high resistance to *R. solani*, as validated across multiple locations and years. Lemont (LE), a japonica rice cultivar from Louisiana, USA, demonstrates high susceptibility to ShB [20,21].

### 4.2. Fungal Inoculation

The highly virulent *Rhizoctonia solani* AG1-IA isolate, YN-7, was used for inoculation [21]. The strain was initially cultured on potato dextrose agar (PDA, Solarbio, Beijing, China) at 28 °C for 3 days. For inoculum preparation, uniform mycelial discs (ca. 0.7 cm in diameter) were transferred into potato dextrose broth (PDB, Solarbio, Beijing, China) containing sterilized wood veneers (1.0 cm × 0.3 cm × 0.8 mm). The cultures were incubated at 28 °C until the veneers were fully colonized by fungal mycelia.

Rice plants were initially grown under natural field conditions in Yangzhou (Jiangsu, China). At the late tillering stage, healthy individuals were selected and transplanted to a long plastic basin in a greenhouse with controlled humidity (45–60%) and temperature (30 °C/13 h, 26 °C/11 h). For inoculation, one mycelia-covered veneer was inserted into the inside of the 2nd leaf sheath from the top of each plant. Subsequently, sheath segments, including 1 cm above and 1 cm below the inoculation site, were harvested at 0 and 24 h post-inoculation (hpi). Mock-treated plants received a sterile, non-colonized wood veneer of the same dimensions, inserted into the corresponding leaf sheath using the same procedure. For each time point and genotype, samples were collected from three independent biological replicates.

### 4.3. Identification of SCPL Gene Family in Rice

The rice reference genome assembly IRGSP-1.0 and the corresponding Ensembl Plants release 54 annotation (https://plants.ensembl.org/, accessed on 21 October 2025) were used for *OsSCPL* identification and subsequent analyses. A representative proteome was constructed by retaining the longest annotated protein sequence at each stable gene locus. Arabidopsis SCPL protein sequences and a previously cloned rice SCPL protein [13] were used as queries in BLASTP searches [22] against the rice representative proteome using NCBI BLAST+ v2.17.0 (National Center for Biotechnology Information, Bethesda, MD, USA). Hits were retained when the E-value was <1 × 10^−5^, sequence identity was >35%, and query coverage per subject (qcovs) was >50%. The coverage criterion referred to the BLAST-reported query coverage across all high-scoring segment pairs (HSPs) for a given query–subject pair.

The entire representative proteome was independently searched using HMMER v3.3 (Sean R. Eddy Laboratory, Harvard University, Cambridge, MA, USA) [23] and the Pfam Peptidase S10 profile PF00450.29 (https://www.ebi.ac.uk/interpro/entry/pfam/PF00450/, accessed on 25 October 2025). Model-specific per-sequence and per-domain gathering thresholds were applied using the -cut_ga option. The final primary analysis set comprised 45 nonredundant *OsSCPL* loci supported by both the BLASTP screening criteria and PF00450 matches satisfying these thresholds. Domain completeness was not imposed as a separate retention criterion. Their model-span coverage was calculated as (hmm_to − hmm_from + 1)/420 × 100, where 420 is the PF00450.29 model length, and their annotated protein isoforms were examined using the release 54 peptide dataset.

### 4.4. Physicochemical Properties and Subcellular Localization

The molecular weight (MW), isoelectric point (pI), instability index, aliphatic index, and grand average of hydropathicity (GRAVY) of the SCPL proteins were calculated using the ExPASy ProtParam tool (https://web.expasy.org/protparam/) (accessed on 25 October 2025). Subcellular localization was predicted using the BUSCA server (http://busca.biocomp.unibo.it/) (accessed on 25 October 2025) [24]. Signal peptides were predicted using SignalP 6.0 (https://services.healthtech.dtu.dk/services/SignalP-6.0/) (accessed on 25 October 2025) with the Eukarya model, and transmembrane domains were predicted using the DeepTMHMM web server (https://services.healthtech.dtu.dk/services/DeepTMHMM-1.0/) (accessed on 2 November 2025) with default settings.

### 4.5. Gene Structure and Conserved Motif Analysis

The exon–intron structures of *SCPL* genes were extracted from the genome annotation files [25,26]. Conserved motifs were identified using MEME (https://meme-suite.org/meme/) (accessed on 10 November 2025) with the parameters -mod anr -protein -nmotifs 10 -minw 6 -maxw 200, allowing any number of motif occurrences per protein sequence [27]. The visualization of motif composition and gene structure was performed using TBtools v2.322 (South China Agricultural University, Guangzhou, Guangdong, China) [28].

### 4.6. Phylogenetic Analysis

Sequence alignments of SCPL proteins from rice, *Arabidopsis thaliana*, *Glycine max*, and *Zea mays* were performed using MUSCLE v5.3 [29] (University of California, Berkeley, CA, USA). Alignments were trimmed using trimAl v1.5.rev1 with the automated1 option, retaining 261 and 286 positions for the multispecies and rice-only datasets, respectively. Maximum-likelihood trees were constructed using IQ-TREE v2.4.0 [30] (Center for Integrative Bioinformatics Vienna (CIBIV), University of Vienna, Vienna, Austria). ModelFinder selected Q.pfam+I+R6 and Q.pfam+I+G4 as the respective best-fit models according to the Bayesian information criterion. Branch support was assessed using 1000 ultrafast bootstrap replicates with additional NNI optimization. Untrimmed alignments were also analyzed to assess the sensitivity of the results to trimming. Trees were visualized using iTOL (https://itol.embl.de/) (accessed on 2 November 2025).

### 4.7. Chromosomal Localization and Gene Duplication Analysis

*SCPL* gene positions were mapped onto the 12 rice chromosomes using genome annotation data. An all-versus-all BLASTP search was performed using BLAST+ v2.17.0 with an E-value threshold of ≤1 × 10^−10^ and a maximum of six target sequences per query. After removing self-hits and retaining the best hit per query–subject pair, up to five non-self hits per query were retained. Collinear blocks were identified using MCScanX [31] with the following parameters: -k 50 -g -1 -s 5 -e 1 × 10^−5^ -m 25 -w 5 -b 0. Tandem duplicates were defined as homologous genes immediately adjacent on the same chromosome, with no intervening genes in the analyzed protein-coding gene set. Tandem clusters comprised genes connected by consecutive tandem pairs. Segmental duplicate pairs were identified as direct anchor pairs within MCScanX collinear blocks. Interspecies collinearity between rice and *Arabidopsis thaliana*, *Glycine max*, and *Zea mays* was analyzed using MCScanX. Ka and Ks values and their ratios were calculated using KaKs_Calculator v3.0 [32] to assess selection pressure.

### 4.8. Cis-Element Analysis in Promoter Regions

For each *OsSCPL* gene, the annotated 5′ end of the selected representative transcript was used as an operational transcription start site (TSS). The 2000-bp genomic region immediately upstream of this position was defined as the promoter region. For positive-strand genes, the interval from TSS − 2000 to TSS − 1 was extracted; for negative-strand genes, the interval from TSS + 1 to TSS + 2000 was extracted and reverse-complemented. Complete 2-kb intervals were retained even when they overlapped adjacent annotated genes, to apply a uniform sequence window to all *OsSCPL* genes. *Cis*-regulatory motifs within these sequences were predicted using PlantCARE database (https://ngdc.cncb.ac.cn/databasecommons/database/id/5261) (accessed on 2 November 2025) [33].

### 4.9. RT-qPCR

For each genotype, pathogen-inoculated (TR) and mock-treated (CK) plants were sampled at 0 and 24 h, with three independent biological replicates for each genotype, treatment, and sampling time. Sheaths were harvested at indicated time points in 1.5 mL RNase-free centrifuge tubes and immediately frozen in liquid nitrogen. Total RNA was extracted using TRIzol reagent (Invitrogen, Carlsbad, CA, USA), and Residual genomic DNA was removed using the gDNA wiper supplied with the HiScript II 1st Strand cDNA Synthesis Kit (+gDNA wiper) (R212-01; Vazyme, Nanjing, China). First-strand cDNA was synthesized from 1 μg of total RNA using the same kit according to the manufacturer’s instructions.

RT-qPCR was performed using ChamQ Universal SYBR qPCR Master Mix (Q711; Vazyme, Nanjing, China) on a CFX96 Real-Time PCR Detection System (Bio-Rad Laboratories, Hercules, CA, USA). The amplification program consisted of an initial denaturation at 95 °C for 30 s, followed by 40 cycles of 95 °C for 10 s and 60 °C for 30 s. Fluorescence signals were collected during the annealing/extension step. Gene-specific primers for the 45 *OsSCPL* genes are listed in Supplementary Table S1. Within each genotype, differences between inoculated and time-matched mock-treated samples at 24 h were evaluated using two-sided Welch’s *t*-tests based on the ΔCt values of three independent biological replicates.

## 5. Conclusions

In this study, 45 serine carboxypeptidase-like (*OsSCPL*) genes were identified in rice using an updated genome annotation, refining the previously reported 71-candidate dataset and providing a more reliable basis for comparative studies. Evolutionary analyses revealed that tandem and segmental duplications contributed to family expansion, with predominantly purifying selection among the analyzed homologous gene pairs. Mock-controlled RT-qPCR analysis identified 14 *OsSCPL* genes responsive to *R. solani* AG1-IA inoculation in at least one of the two rice genotypes, highlighting *OsSCPL4* and *OsSCPL25* as candidates for further investigation. These findings provide an updated genomic resource for rice SCPL research and a basis for investigating the molecular responses of rice to the sheath blight pathogen. Although the roles of these candidate genes in disease resistance require further functional validation, this study may facilitate the identification of genetic resources for resistance breeding and inform future strategies for sustainable sheath blight management.

## Data Availability

The original contributions presented in this study are included in the article/Supplementary Material. Further inquiries can be directed to the corresponding author.

## Supplementary Materials

The following supporting information can be downloaded at: https://www.mdpi.com/article/10.3390/ijms27219850/s1.

## Abbreviations

The following abbreviations are used in this manuscript:

ABRE	Abscisic acid-responsive element
ACC	1-Aminocyclopropane-1-carboxylic acid
ARE	Anaerobic-responsive element
CK	Time-matched mock-treated control
DRE	Dehydration-responsive element
ERE	Ethylene-responsive element
hpi	Hours post-inoculation
HSPs	High-scoring segment pairs
JA	Jasmonic acid
MBS	MYB-binding site
PDA	Potato dextrose agar
PDB	Potato dextrose broth
RT-qPCR	Reverse transcription quantitative polymerase chain reaction
SA	Salicylic acid
SCP	Serine carboxypeptidase
SCPL	Serine carboxypeptidase-like
ShB	Sheath blight
STRE	Stress-responsive element
TR	*Rhizoctonia solani*-inoculated treatment
